# Interaction of drought‐ and pathogen‐induced mortality in Norway spruce and Scots pine

**DOI:** 10.1111/pce.14360

**Published:** 2022-05-31

**Authors:** Mireia Gomez‐Gallego, Lucia Galiano, Jordi Martínez‐Vilalta, Jan Stenlid, Hernán D. Capador‐Barreto, Malin Elfstrand, J. Julio Camarero, Jonàs Oliva

**Affiliations:** ^1^ Department of Forest Mycology and Plant Pathology Swedish University of Agricultural Sciences Uppsala Sweden; ^2^ Université de Lorraine, INRAE, IAM Nancy France; ^3^ CREAF, Bellaterra (Cerdanyola del Vallès) Catalonia Spain; ^4^ Universitat Autònoma de Barcelona, Bellaterra (Cerdanyola del Vallès) Catalonia Spain; ^5^ Pyrenean Institute of Ecology (IPE‐CSIC) Zaragoza Spain; ^6^ Department of Crop and Forest Sciences University of Lleida Lleida Spain; ^7^ Joint Research Unit CTFC‐AGROTECNIO Lleida Spain

**Keywords:** carbon starvation, conifer, drought‐induced tree death, hydraulic failure, necrotrophic pathogen, sapwood

## Abstract

Pathogenic diseases frequently occur in drought‐stressed trees. However, their contribution to the process of drought‐induced mortality is poorly understood. We combined drought and stem inoculation treatments to study the physiological processes leading to drought‐induced mortality in Norway spruce (*Picea abies*) and Scots pine (*Pinus sylvestris*) saplings infected with *Heterobasidion annosum* s.s. We analysed the saplings' water status, gas exchange, nonstructural carbohydrates (NSCs) and defence responses, and how they related to mortality. Saplings were followed for two growing seasons, including an artificially induced 3‐month dormancy period. The combined drought and pathogen treatment significantly increased spruce mortality; however, no interaction between these stressors was observed in pine, although individually each stressor caused mortality. Our results suggest that pathogen infection decreased carbon reserves in spruce, reducing the capacity of saplings to cope with drought, resulting in increased mortality rates. Defoliation, relative water content and the starch concentration of needles were predictors of mortality in both species under drought and pathogen infection. Infection and drought stress create conflicting needs for carbon to compartmentalize the pathogen and to avoid turgor loss, respectively. *Heterobasidion annosum* reduces the functional sapwood area and shifts NSC allocation patterns, reducing the capacity of trees to cope with drought.

## Summary Statement

Interrelated decreases in carbon and water pools drive drought‐ and pathogen‐induced mortality mechanisms in *Picea abies* infected by the pathogen *Heterobasidion annosum*. The pathogen reduces the functional sapwood area and induces a defence response, reducing available carbon to face drought impacts.

## 1 INTRODUCTION

Global change is posing increasing challenges for forest ecosystems. Emergent and/or invasive pests and diseases can be the primary cause of extensive tree death (e.g., Anagnostakis, 1987; Díaz‐Yáñez et al., 2020; Rizzo et al., 2002). Diseases not only emerge following the introduction of pathogens to new environments but also by modifying their virulence due to climate‐driven processes (Ghelardini et al., 2016). Therefore, it is increasingly important both to predict the risk of an acceleration in the development of existing diseases linked to climate change and to understand pathogen‐induced tree death. In turn, drought is a well‐recognized driver of large‐scale tree mortality and long‐term ecosystem dynamics (Allen et al., 2010, 2015; Brodribb et al., 2020; Hartmann et al., 2018). Although drought‐induced tree mortality has been intensively studied over the past two decades (for a review see Adams et al., 2017; Choat et al., 2018), the pathways leading to tree mortality are not completely understood yet. Often tree mortality arises from an interaction between biotic (pests and pathogens) and abiotic stress agents, rather than being the result of a single disturbance factor (Millar & Stephenson, 2015; Schoeneweiss, 1986; Simler‐Williamson et al., 2019; Stephenson et al., 2019). This adds complexity to our understanding of mortality pathways, and hence, modelling mortality and future forest dynamics remains challenging (Bugmann et al., 2019; Trugman et al., 2021).

Tree mortality under drought conditions may be a consequence of hydraulic failure, the exhaustion of carbohydrate reserves, impaired phloem transport or a combination of these factors (Adams et al., 2017; Choat et al., 2018; Martinez‐Vilalta et al., 2019; Salmon et al., 2019). In the presence of severe drought, dramatic hydraulic failure occurs, with tree mortality typically associated with a loss of hydraulic conductivity of at least 60% (Adams et al., 2017); however, the mechanistic link remains to be established (Mantova et al., 2021). An impaired conductive system interrupts the soil–root hydraulic continuum, which jeopardizes the delivery of sufficient water to the canopy, leading to critical cell dehydration (Körner, 2019; Martinez‐Vilalta et al., 2019). Carbohydrates are required for osmoregulation to avoid turgor loss and may have a relevant role in maintaining the tree's water status under declining water availability (Sapes et al., 2019; Sevanto et al., 2014). Even within a species, the role of NSCs in the pathway to mortality may vary depending on the tree's strategy for mitigating drought stress, with some trees suffering fast, catastrophic hydraulic failure but unaltered carbohydrate reserves, while others show a clear contribution of hydraulic constraints and carbohydrate use in the mortality process (Sevanto et al., 2014).

When necrotrophic fungi infect the stem or roots, they can destroy the cambium and vascular tissue of the infected host, potentially inducing hydraulic impairment and affecting carbon (C) and water transport in the tree (Oliva et al., 2014). Trees defend their sapwood from necrotrophic fungal pathogens by forming C‐expensive barriers (Guérard et al., 2007), resulting in areas of nonconducting sapwood. As stated by Alex L. Shigo (1984): 'Trees do not cast off dead and dying parts. Trees form boundaries between dying parts and the healthy frame of the wood and the inner bark'. In the sapwood, an injury or an infection may be compartmentalized by the formation of a reaction zone (Pearce, 1996). In the reaction zone, the sapwood is both physically and chemically modified, involving de novo synthesis of secondary metabolites from NSCs (Oliva et al., 2012), which relies on the availability of C at the site of infection (Guérard et al., 2007). When drought coincides with pathogens or pest attacks, the role of C depletion in the pathway to mortality is thought to be amplified (Gaylord et al., 2013, 2015; McDowell et al., 2008; Oliva et al., 2014). On the one hand, drought‐induced stomatal closure decreases C uptake. On the other hand, C may be allocated to distal tissues suffering pathogen infection. Thus, in addition to the pathogen‐induced loss of sapwood, the increased C demand for defence reactions can potentially accelerate drought‐induced tree death in the case of necrotrophic pathogens infecting the stem or roots. However, there is little empirical evidence to support these hypotheses.

The necrotrophic pathogenic fungi of the *Heterobasidion annosum* sensu lato species‐complex cause root and butt rot in most species of conifers trees in the northern hemisphere (Gonthier et al., 2013). Both *Heterobasidion parviporum* and *Heterobasidion annosum* sensu stricto, two of the European species that belong to this complex, are strong pathogens on Norway spruce (*Picea abies* (L.) Karst.). *Heterobasidion parviporum* is specialized in Norway spruce, whereas *H. annosum* s.s. is usually associated with Scots pine (*Pinus sylvestris* L.) but can also infect and damage Norway spruce (Garbelotto & Gonthier, 2013). The species in this complex primarily enter the tree via wounds and colonize the sapwood of living trees. Conifers differ in the way that they respond to these pathogens. In Norway spruce, *H. annosum* s.s. attacks the sapwood and colonizes the heartwood of the root system and the lower part of the stem. From the inner sapwood, the pathogen spreads outwards and can reach and damage the phloem. Norway spruce compartmentalizes the pathogen by creating a reaction zone impregnated with secondary metabolites (Oliva et al., 2012). By contrast, in Scots pine, the pathogen cannot colonize the heartwood and occurs only in the sapwood and the phloem (Korhonen & Stenlid, 1998). Scots pine typically shows a weaker defence response to infection by *H. annosum* s.s. than spruce (Nagy et al., 2006), and the reaction zone may include resin inclusions and discolouration but is not always visible (Wang et al., 2014). Analyses of the interaction between *H. annosum* s.l. and drought in conifers are scarce. *Heterobasidion parviporum* has been suggested to increase drought stress in Norway spruce (Gori et al., 2013). In addition, drought has been shown to lead to larger necrotic lesions in trees inoculated with either *H. parviporum* or *H. annosum* s.s. compared with nondrought conditions (Terhonen et al., 2019). However, the effect of drought on the development of *H. annosum* s.s. decay in the sapwood and the effects of these combined stresses on the mortality process in Norway spruce and Scots pine remain to be tested. The drought responses of both tree species have been described. They both present strict stomatal control and progressive leaf loss under limiting water availability (Havranek & Benecke, 1978; Jamnická et al., 2019; Poyatos et al., 2013). Therefore, the two tree species, together with their shared pathogen *H. annosum* s.s., provide a suitable system for studying the interaction between drought and necrotrophic pathogens.

Here, we used the host species Norway spruce and Scots pine and the pathogen *H. annosum* s.s. as models to study the interaction between pathogenic infection and drought as drivers of tree mortality in conifers, focusing on the effects of these stressors on the C and water balance of saplings. We tested the following Hypotheses: (i) that the interaction between drought and infection by a necrotrophic pathogen has an additive effect and increases the risk of mortality compared with either stressor alone; (ii) this increased mortality risk is due to the three following nonexclusive, interactive processes: (1) drought reduces C reserves and impairs the capacity of trees to build up defence barriers to compartmentalize the pathogen; (2) defence needs associated with pathogenic infection reduce the amount of C available to cope with drought, which eventually impairs water status; and (3) pathogen infection affects hydraulic performance due to a reduction in the sapwood cross‐sectional area.

## 2 MATERIALS AND METHODS

### 2.1 Plant material and experimental design

A total of 46 *P. abies* (L.) Karst. and 47 *P. sylvestris* L. open‐pollinated saplings that had been grown in a field at a commercial nursery and produced following standard procedures (Härnevi Nursery) were transferred to 40‐L pots and grown in a thermostatically controlled greenhouse (SLU) from May to August 2017. The air temperature fluctuated between 16°C and 30°C during the day and between 8°C and 18°C during the night, depending on the weather conditions. Saplings were 6 years old and 1.5–2.0 m tall with a mean diameter of ca. 5 cm (at 10 cm from the collar). On 22 August 2017, half of the saplings were inoculated by inserting infected wood plugs into holes drilled in the trunk, which were then sealed with silicon. Six 5‐mm holes 2‐cm apart were drilled in each sapling. Inoculum was produced by placing autoclaved wood plugs in sealed Petri dishes colonized with the *H. annosum* s.s. isolate Mj 87, which has been shown to be highly pathogenic on both Norway spruce and Scots pine in previous trials (Swedjemark et al., 1999; *H. annosum* s.s. is referred to as the P‐intersterility group of *H. annosum* s.l. in this publication). The Petri dishes were incubated at 20°C in the dark. The other half of the saplings served as a control and were treated by inserting sterile plugs into the holes. The saplings were watered once a week both before (May–August 2017) and during the experiment (August 2017–August 2018). Two different watering regimes were used corresponding to a gravimetric soil water content of ca. 20% (drought) or 40% (well‐watered). The resulting treatment combinations were: well‐watered/noninoculated (W/NI; *N* = 10 for spruce and 7 for pine); well‐watered/inoculated (W/I; *N* = 9 for each species); drought/noninoculated (D/NI; *N* = 10 for spruce and 8 for pine); and drought/inoculated (D/I; *N* = 10 for spruce and 9 for pine). Soil moisture was assessed the day before watering with a Decagon GS1 sensor coupled to a ProCheck Handheld Reader. Soil moisture was measured in mV and was converted to volumetric soil water content (*VWC*) by a calibrated curve obtained from soil samples with known *VWC*. Transformation from *VWC* to pF (i.e., the logarithm of the absolute value of the soil matric potential) and then to water potential was obtained from a characteristic soil curve conducted by the METER Group AG.

The growing conditions during the experiment (from 22 August 2017 until 2 August  2018) were 16 h of light at 22°C and 8 h of dark at 18°C. We also simulated a 10‐week dormancy period with the following conditions: 3 weeks at temperatures between 10°C and 15°C and no light from 11 December 2017 to 2 January 2018; 6 weeks at 5°C and no light from 3 January to 18 February 2018; and 1 week with light and temperatures between 10°C and 15°C from 19 to 26 February 2018 (Supporting Information: Figure S1).

### 2.2 Physiological measurements

The saplings' physiological status and defoliation were monitored once before performing the treatments and then from 17 October 2017, onwards, that is, 2 months after the treatments were performed, and then every 2 weeks for a year. During the 10‐week dormancy period, only two measurements were made (see Supporting Information: Figure S1 for an overview of measurements). We measured the net assimilation rate (*A*), stomatal conductance (*g*_s_) and transpiration rate (*E*) on fully expanded current‐year needles using an LI‐COR LI‐6400XT gas‐exchange analyser system (LI‐COR). Chamber conditions were kept at 400 μmol CO_2_ m^−2^ s^−1^, 1700 μmol photons m^−2^ s^−1^, 40% relative humidity and 28°C block temperature. Measurements were taken once steady‐state gas exchange was achieved. Instantaneous photosynthetic *WUE* was calculated as the ratio *A*/*E*. In addition, we assessed defoliation by assigning a grade from 0 to 4 (0, no defoliation; 1, 25% defoliated; 2, 50% defoliated; 3, 75% defoliated; 4, completely defoliated). We considered defoliation caused by the treatments to be any excess defoliation relative to the control treatment. Hence, we interpreted that a treatment caused defoliation when this was significantly higher than that of the control treatment. To analyse the risk of mortality associated with each treatment and species (Hypothesis i), we also recorded mortality every 2 weeks based on complete defoliation, cambial death (presence of necrotic cambium after removing the bark) and extremely low (undetectable) gas exchange.

On each sampling date, a branch at mid‐height on each sapling was chosen. One twig on this branch was used to measure midday water potential (*Ψ*_md_) and a fascicle was sampled to measure the needle relative water content (*RWC*) (see Supporting Information: Figure S1 for sampling dates). In addition, one spruce twig was sampled on four sampling dates and one pine twig was sampled on two sampling dates to determine the NSC level of a fascicle. One spruce and one pine twig were also sampled on two dates to obtain measurements of xylem hydraulic conductivity (*K*_h_) and native embolism. For the *RWC* measurements, needles were first placed in vials on ice and weighed in the laboratory to obtain the fresh weight and then rehydrated overnight at 4°C in darkness, blotted dry on a paper towel and weighed again to obtain the turgid weight. Finally, needles were dried in the oven at 70°C for 24 h and reweighed to obtain the dry weight. The *RWC* was calculated as: (fresh weight − dry weight)/(turgid weight − dry weight) × 100. For *Ψ*_md_ measurements, sampled twigs were placed immediately in plastic bags and stored in a cold container until they were measured (within 1–2 h) using a Scholander‐type pressure chamber (Digital 80 Bar Portable Plant Moisture System; Skye Instruments Ltd.).

Xylem conductivity and native embolism were measured twice: before treatments were applied and 3.5 months after treatment. For *K*_h_ measurements, twigs were placed inside plastic bags with a damp cloth, which were then placed in a cooler. In the laboratory, twig segments were recut underwater to obtain a segment of ca. 10 cm with no lateral branches. We debarked the proximal end and put it in a degassed perfusion solution (10 mM KCl and 1 mM CaCl_2_ in ultrapure filtered water), which was placed ca. 50 cm above the segment to generate a pressure gradient of ca. 5 kPa, and let water flow for ca. 10 min. We collected and weighed the solution at the distal end for the next 10 min and then repeated this procedure until consecutive measurements changed by less than 20%. We then calculated *K*_h_ (m^4^ MPa^–1^ s^–1^) as the water flow multiplied by the segment length divided by the pressure gradient. We rehydrated the samples for 48 h and then repeated the procedure to determine the maximum hydraulic conductivity (*K*_h, max_), and sapwood‐specific maximum conductivity (*K*_s, max_) by dividing *K*_h, max_ by the cross‐section. We calculated the percentage loss of conductivity (*PLC*) as 100 × (1 − *K*_h_/*K*_h, max_).

### 2.3 NSC measurements

NSC measurements were only performed on living saplings. Each sampled twig and its current‐year needles (see Section 2.2 for details of twigs used for NSC sampling) were microwaved immediately after sampling for 90 s at 600 W to stop the enzymatic activity and then subsequently oven‐dried for 72 h at 70°C. Spruce stem and root samples were taken from surviving trees at the end of the experiment. Stem samples were obtained by cutting out a ca. 1 cm^3^ piece of sapwood ca. 8 cm above the uppermost hole of the inoculation treatments. Root samples were obtained by cutting off pieces of roots that were less than 0.5 cm in diameter. Stem and root samples were immediately frozen until processed. All samples were milled in a ball mill (Retsch M400) to obtain a fine and homogeneous powder. Soluble sugars (SS) were extracted with 80% (v/v) ethanol in a water bath at 60°C and their concentration was determined colorimetrically as described by Buysse and Merckx (1993). Starch and complex sugars remaining in the undissolved pellet after ethanol extractions were enzymatically reduced to glucose using 0.5% amyloglucosidase (Fluka 10115) and analysed following Palacio et al. (2007). NSCs measured after ethanol extraction were considered to be SS and carbohydrates measured after enzymatic digestion were considered to be starch. Both components were expressed in glucose equivalents. The total NSC concentration of a sample was considered to be the sum of SS and starch.

Our final NSC data set for pine comprised measurements of needle SS and starch concentrations before and 2 months after the onset of treatments (17 October 2017) (see Supporting Information: Figure S1 for sampling dates). For spruce, we measured the SS and starch concentrations of needles before treatments, 2 and 3 months after treatments (17 October and 15 December 2017) after dormancy (6 months after treatments, 28 February 2018), and in twigs before and after dormancy (15 December 2017 and 28 February 2018). In addition, the SS and starch concentrations of needles, stems and roots of spruce survivors were measured 1 year after the onset of treatments (2 August 2018) involving individual stressors or the control treatment. The NSC data set did not include these measurements for pine survivors because the sample size was not large enough (less than three living saplings survived in each treatment group: W/I, D/NI and D/I).

### 2.4 Infection assessments

We monitored lesion length 2 and 3 months after treatments were performed (see Supporting Information: Figure S1). The outer bark was slightly scratched to reveal the colonized phloem tissue. The radius of the ellipsoid‐shaped necrosis was measured horizontally in all spruce saplings. Most of the inoculated pine saplings were early girdled, and hence, we did not monitor lesion length in pine saplings.

To test the relationship between C stores and the defence reaction in spruce sapwood, which involves the formation of a reaction zone to try to compartmentalize the infection, we analysed three cross‐sections of the treated part of the stem (i.e., the top, middle and bottom of the inoculated section) per sapling (Supporting Information: Figure S2). We sprayed these sections with the pH indicator 2,6 dichlorophenolindophenol to discriminate the reaction zone (tissues with a pH above 6.0 were dyed blue) from decayed zones (tissues with a pH of less than 5.5 were dyed red) (Johansson & Theander, 1974; Liu et al., 2020; Oliva et al., 2015). Dyed and undyed images were used to calculate the proportion of each section corresponding to functional sapwood, decayed sapwood, reaction zone and active reaction zone based on pixel counts after manual classification using Adobe Photoshop 21.2.4 (Adobe Systems Inc.). In some cases, decayed sapwood was clearly divided into two consecutive colonization episodes, and thus, was recorded as either intermediate or advanced decayed sapwood. Heartwood was absent in all saplings. Cross‐section measurements were only performed for spruce after death or at the end of the experiment in surviving saplings. Spruce saplings that received the ‘D/I' treatment were not included in these analyses because none of these saplings was still alive by the end of the experiment.

### 2.5 Data analyses

We used accelerated failure time models, which can deal with censored data (i.e., the presence of samples that die at different time points as well as samples that are alive at the end of the experiment), to assess the effect of treatment, species and their interaction on survival probability (Hypothesis i). We used a parametric test (function *survreg*, package *survival* in R; Therneau, 2021), which allowed factors and interactions. A log‐logistic error distribution was applied.

To analyse the impact of the treatments on the saplings' physiology, we performed linear mixed models, with the different physiological variables (*RWC*, *Ψ*_md_, *A*, *g*_s_, *WUE*, defoliation, SS and starch in spruce needles) as response variables, ‘water' and ‘inoculation' and their interaction (when significant) as fixed factors, and sampling date as a random factor, which were performed separately for each species (function *lme*, package *nlme* in R; Pinheiro et al., 2021). We modelled variance heterogeneity, when needed, using either a power or a constant power variance function (varPower or varConstPower, package *nlme*). The two sampling dates taken during the simulated winter were excluded from the analyses because the saplings were dormant. We fitted a linear model, instead of a linear mixed model, for the response variables SS and starch in pine needles and for *PLC* in spruce and pine because only one sampling date (17 October 2017) was available after the onset of treatments (hence no need for a random factor). For spruce, we analysed the lesion length, which was measured on two sampling dates (2 and 3 months after treatment onset), as a response variable in a linear mixed model, which included the treatment as a fixed factor and the tree as a random factor.

To study the pathway to mortality caused by each stressor individually and when the two stressors were combined for each species (Hypothesis ii), we compared each of the stress treatments (‘W/I', ‘D/NI' and ‘D/I') to the control (‘W/NI'). To do that, we subset the data for each of those three treatments for each species, including the control treatment in each subset, and fitted different models for each combination. We modelled survival probability through time as a function of different predictor variables (an individual model for each variable). We included in different models either the initial values (before the onset of treatments) or the repeated measurements across the experiment (time‐dependent variables) for each one of the physiological and health‐related variables. We used nonparametric models (function *coxph*, package *survival* in R; Therneau, 2021), which allowed us to model time‐dependent responses. The variables that were included in the models with their initial and time‐dependent values were: *A*, *g*_s_, *E*, *WUE*, *RWC*, *Ψ*_md_, *PLC*, *K*_s, max_, starch and SS concentrations (the latter measured in needles for pine and in twigs and needles for spruce). However, only the initial *SLA* and *K*_h, max_ values and only the time‐dependent values for defoliation and lesion length (only in spruce) were included in the models. A total of 22 models were fitted for each of the three subsets of data for pine, and 26 models for each of the three subsets of data for spruce. The effect of each variable on the survival of saplings was quantified as the number of days by which survival increased per unit increase in the standardized explanatory variable. These values were useful for comparing the magnitude of the impact of each variable.

We studied the mechanisms by which the interaction of drought and pathogen infection would lead to mortality using several additional models. To test the link between the defence response to infection and C expenses for inoculated spruce, we modelled the effect of the total proportion of the reaction zone on the concentration of SS in the stem (we used beta‐regression because we were modelling proportion data), and on the decayed proportion of the cross‐section (we used a linear model because the distribution of this latter variable was normal). To analyse the link between the water status and the NSC content of spruce, we used a linear mixed model with log‐transformed *RWC* as a response variable and log‐transformed needle NSC concentration as a predictor, with the sampling date included as a random factor. We also used a beta‐regression model to explain *RWC* as a function of NSC for spruce, using stem NSC concentrations measured at the end of the experiment and the average *RWC* values across the experiment for each sapling (function *betareg*, package *betareg* in R; Cribari‐Neto & Zeileis, 2010). In addition, we modelled the degree of defoliation, with a linear mixed model as a function of *RWC*, including species, treatment and its interaction as fixed factors, and the sampling date as a random factor. Finally, we analysed whether W/I (and D/NI) spruce survivors and nonsurvivors (and W/NI survivors for reference) presented different levels of SS and starch in needles and twigs before and after dormancy using a beta‐regression model.

## 3 RESULTS

### 3.1 Simultaneous drought and pathogen infection increased tree mortality in Norway spruce

**Figure 1:**
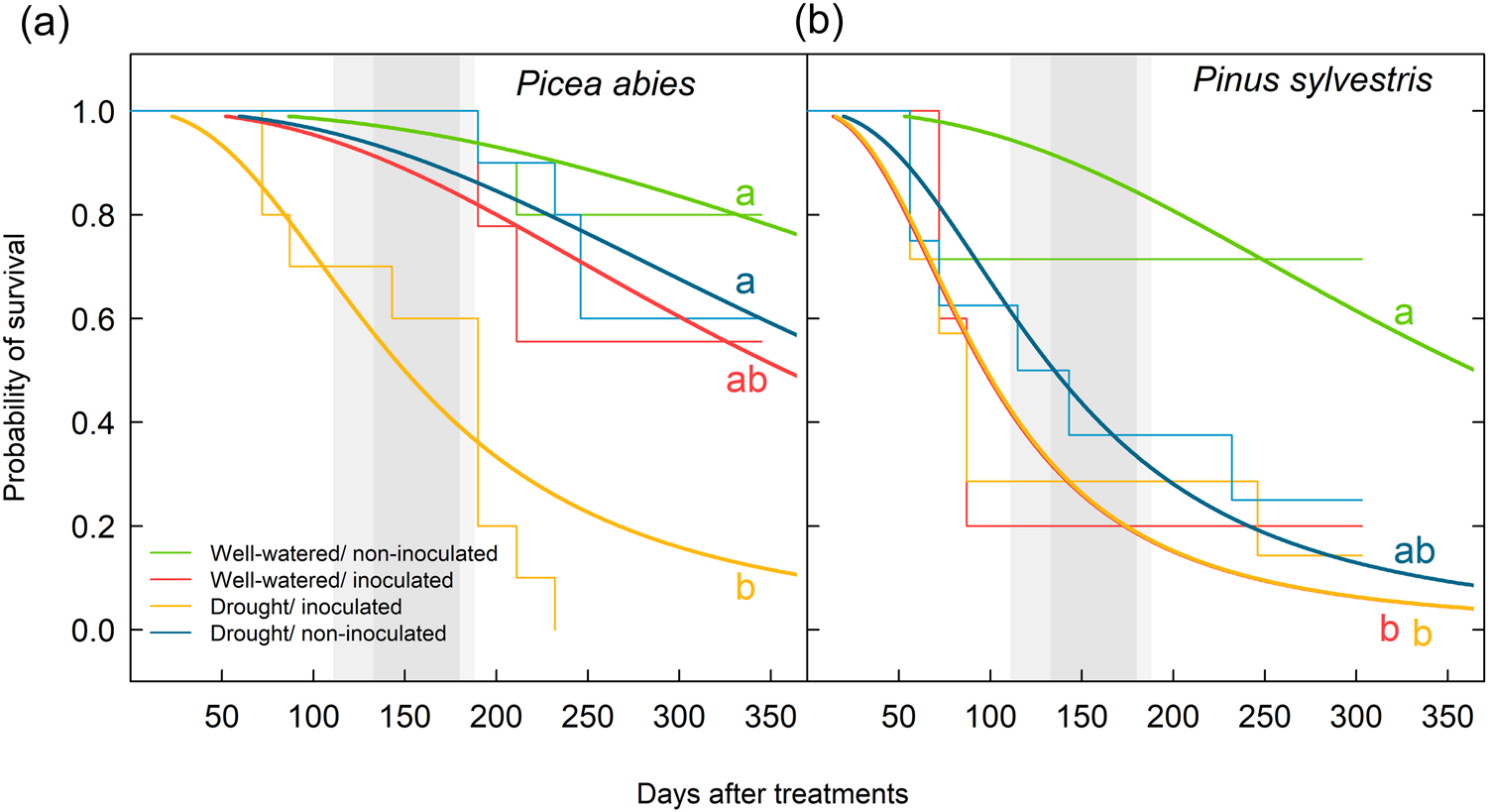
Predicted survival time from the accelerated failure time parametric model fit for each treatment and species. Treatments: W/NI, well‐watered/non‐inoculated; W/I, well‐watered/inoculated with *Heterobasidion annosum* s.s.; D/I, drought/inoculated with *H. annosum* s.s.; D/NI, drought/non‐inoculated. Different letters indicate statistically significant differences between treatments within a species at *p* = 0.05 when performing a multiple comparison procedure using Tukey. Thin lines indicate empirical data. Grey‐shaded areas correspond to the dormancy period. NB: There are marginal differences between W/I and drought/inoculated treatments in spruce (*p* = 0.059).

The combination of drought and pathogen infection posed a higher mortality risk than each individual stressor in spruce. Drought‐inoculated spruce died at a faster rate than spruce saplings that were only subjected to drought or inoculation (marginal difference, *p* = 0.059) (Figure 1a and Supporting Information: Table S1). Inoculated spruce saplings that were subjected to drought suffered mortality sooner than those that received the well‐watered treatment and the mortality rate accelerated after the dormancy period (30% vs. 90% of accumulated deaths before and after dormancy, respectively) (Figure 1a). Spruce saplings subjected to drought alone suffered mortality later than saplings subjected to the other treatments, with the first deaths occurring 6 months after the onset of treatments and peaking in April 2018, coinciding with bud flush (Figure 1a).

**Figure 2:**
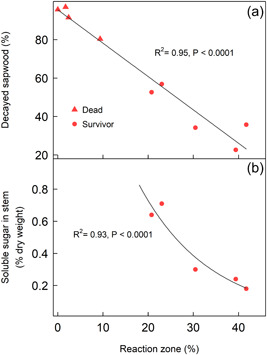
Linear relationship between the total percentage of the reaction zone and the decayed sapwood for dead and surviving spruce (a). Beta‐regression linking the total reaction zone with the concentration of soluble sugars (SS) at the site of infection for surviving spruce at the end of the experiment (b).

Drought influenced the capacity of trees to defend themselves from the pathogen. Two and three months after inoculation, drought‐inoculated saplings showed larger necrotic lesions than well‐watered inoculated saplings (Supporting Information: Figure S3). In spruce, the survival of inoculated saplings at the end of the experiment was associated with the capacity to create a larger reaction zone in the sapwood to compartmentalize the pathogen and to protect the functional sapwood (31% vs. 3% of the cross‐section in surviving and dead saplings, respectively). The size of the reaction zone was inversely correlated with the area colonized by the pathogen, measured as the percentage of the cross‐section with evidence of wood decay (Figure 2a). In surviving spruce saplings, reaction zone size was negatively associated with the SS concentration in the stem (Figure 2b).

**Figure 3:**
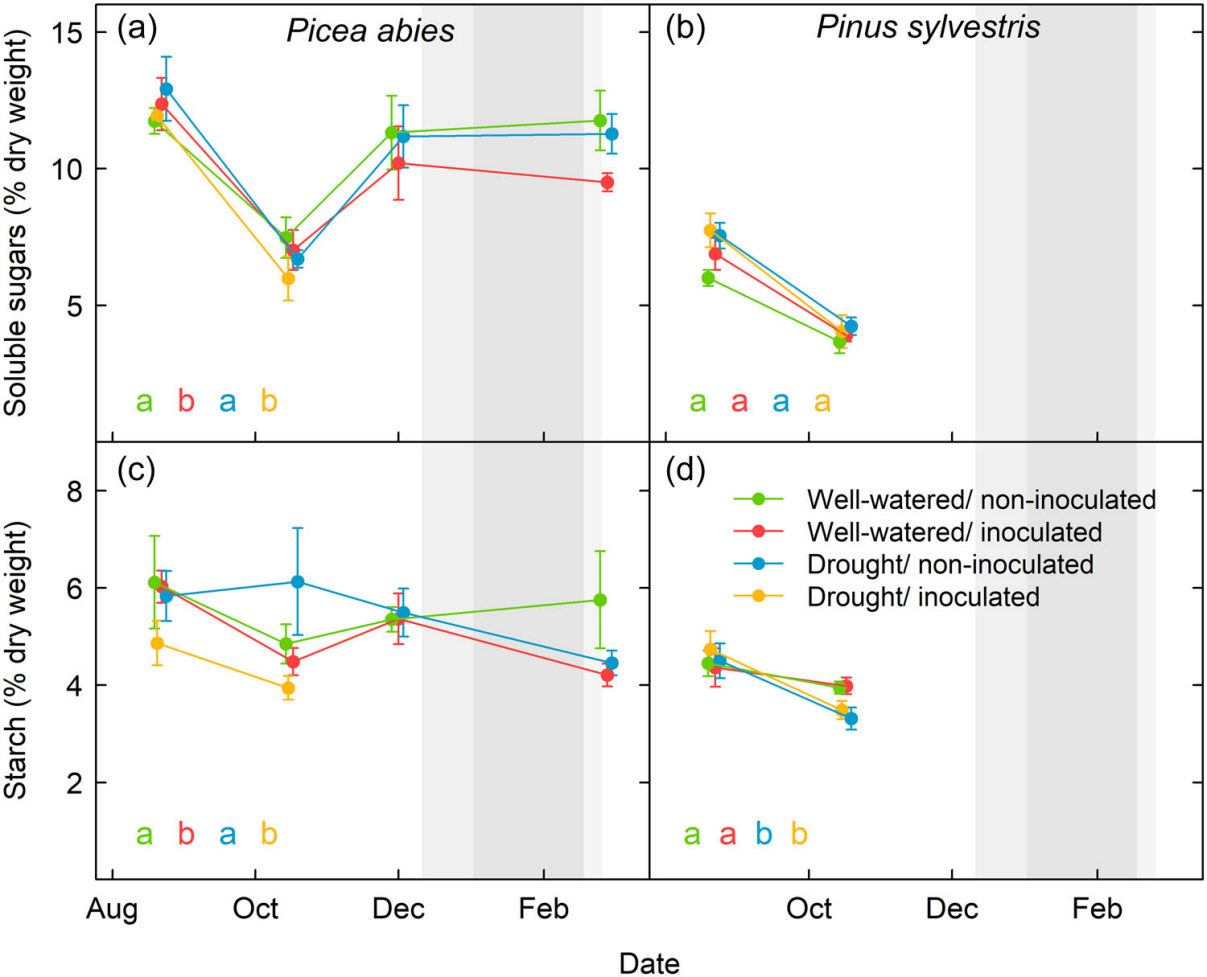
Soluble sugars and starch content (% dry weight) of *Picea abies* (a, c) and *Pinus sylvestris* needles (b, d). Different letters indicate statistically significant differences (*p* = 0.05) between groups when performing a multiple comparison procedure using Tukey. For spruce, mixed models include the three sampling dates. In pine, a linear model is fitted for the unique sampling date after treatments (refer to Section 2.5). Grey‐shaded areas correspond to the dormancy period.

**Figure 4:**
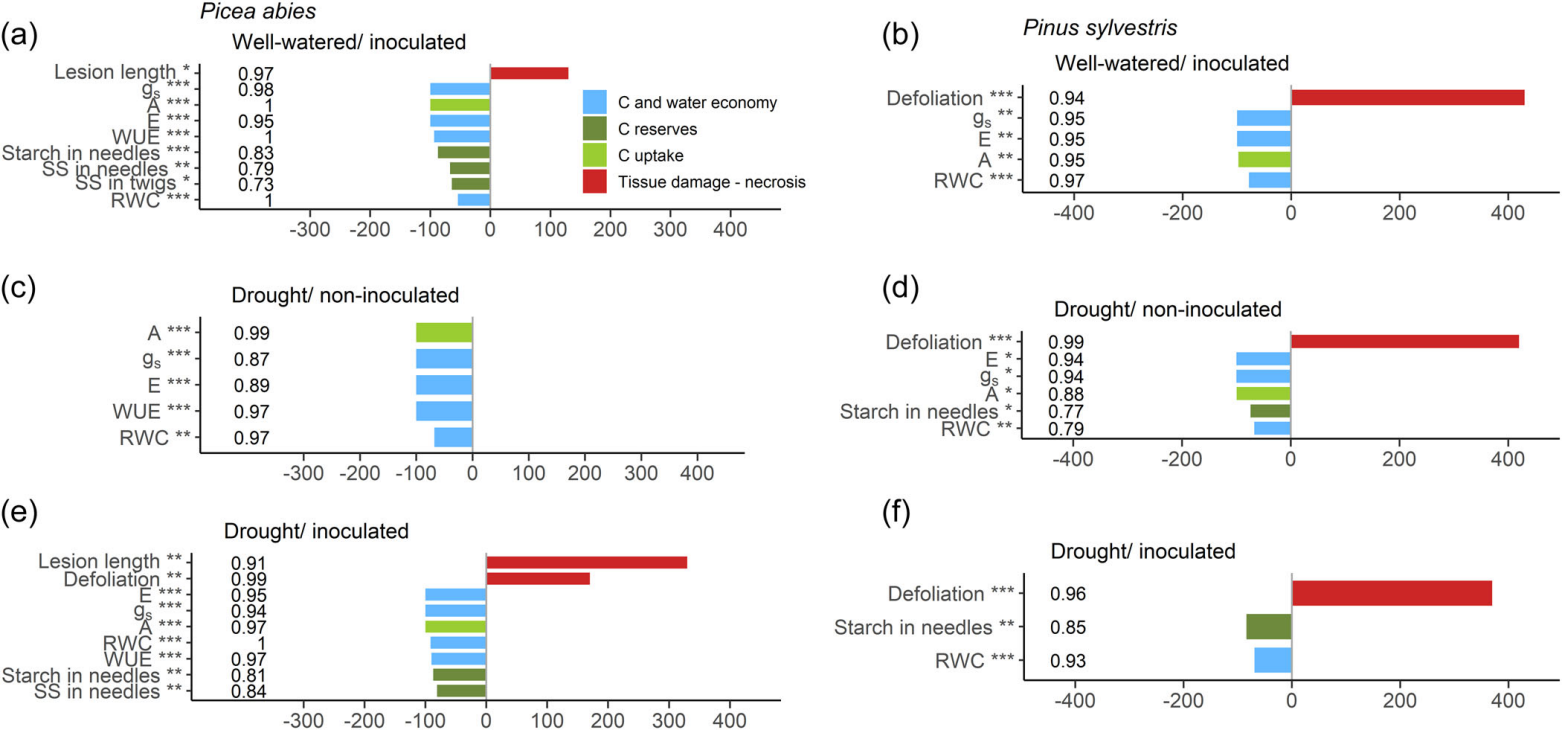
Effect of physiological and health variables on the survival of *Picea abies* (a, c, e) and *Pinus sylvestris* (b, d, f) saplings, quantified as the number of days (*x*‐axis) for which mortality was anticipated (positive) or delayed (negative values) per unit increase in the standardized explanatory variable, as given by the accelerated failure time models. Only significant variables are shown (*p < 0.05, **p ≤ 0.01, ***p ≤ 0.001). Values of concordance of each model are shown next to each explanatory variable. Concordance measures model accuracy as the fraction of pairs for which the model is correct. *A*, net assimilation rate; *E*, transpiration rate; *g*_s,_ stomatal conductance; *Ψ*_md_, midday water potential; *RWC*, needle relative water content; SS, concentration of soluble sugars; starch, concentration of starch; *WUE*, water use efficiency; *Ψ*_md_, midday water potential.

Needles from inoculated spruce saplings presented lower SS and starch concentrations than needles of noninoculated saplings subjected to either water treatment (Figure 3 and Supporting Information: Table S2). Drought did not decrease the concentration of starch and SS in needles (Figure 3a,c and Supporting Information: Figure S4c,d,g,h). Indeed, we found an association between low NSC levels and the mortality of inoculated saplings (regardless of the watering regime), but not under drought in the absence of infection in spruce (Figure 4a,c,e). Starch and SS concentrations in needles were predictors of death for saplings that received either of the two treatments involving pathogen inoculation (Figure 4a,e and Supporting Information: Figure S4 and Table S3).

**Figure 5:**
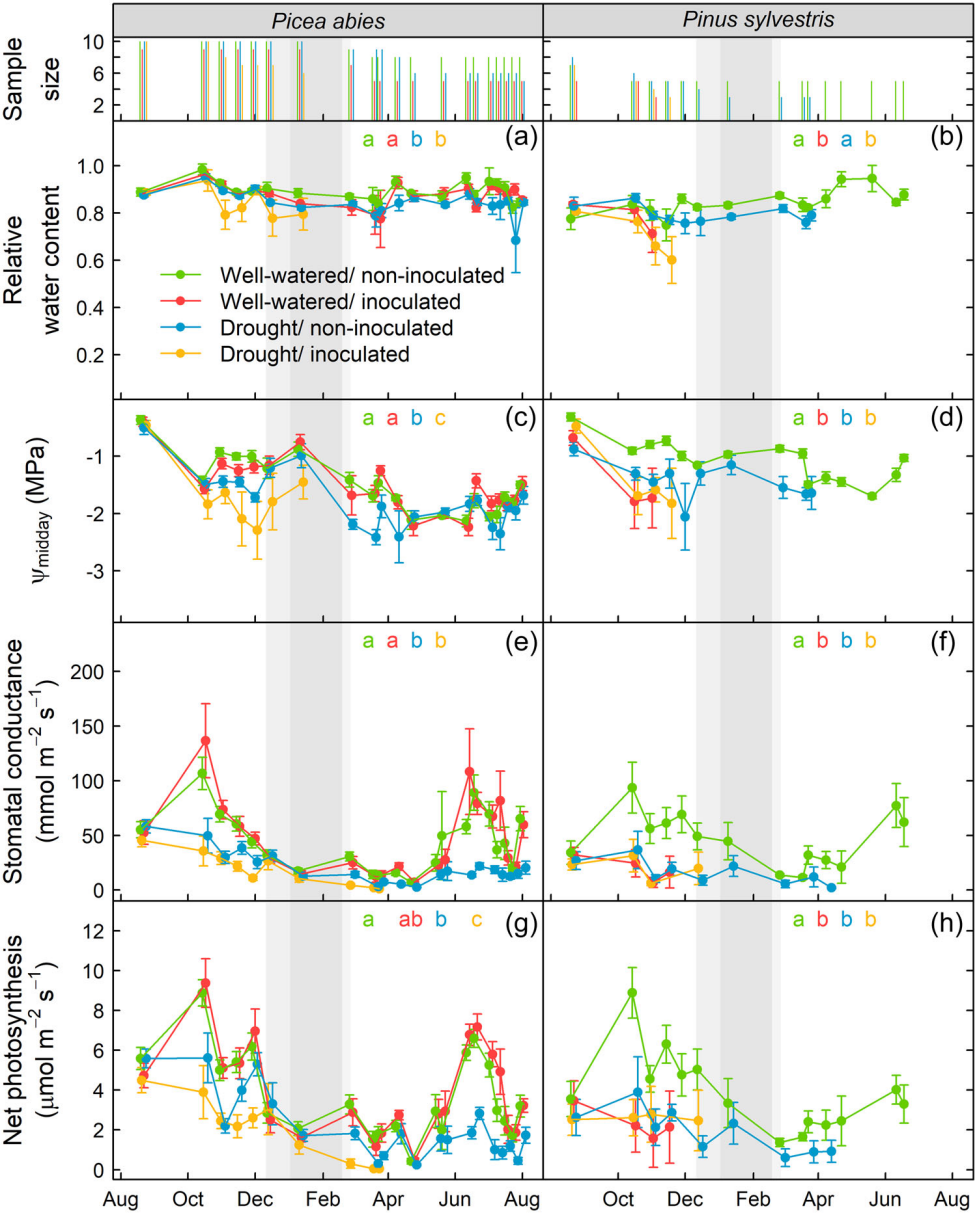
Time series of the physiological variables for each treatment and species: (a, b) relative water content in needles, (c, d) midday water potential, (e, f) stomatal conductance, and (g, h) net photosynthesis. Different letters indicate statistically significant differences (*p* = 0.05) between treatments within species when analysed by linear mixed models when performing a multiple comparison procedure using Tukey. The sample size (number of saplings) per sampling date is indicated in the top panels. Grey‐shaded areas correspond to the dormancy period.

Drought reduced the *Ψ*_md_, *A* and *g*_s_ of saplings (Figure 5) and led to a higher *PLC* 3 months after treatments compared with that of control saplings (Supporting Information: Figure S5). Inoculation alone did not cause any hydraulic impairment, with no increase observed in the *PLC* of xylem in inoculated‐only spruce and all physiological variables were similar to those of controls (*RWC*, *Ψ*_md_, *g*_s_, *A*; Figure 5 and Supporting Information: Figure S5 and Table S2). However, inoculation exacerbated the hydraulic stress seen in saplings subjected to drought given that lower *Ψ*_md_ values were found in drought‐inoculated saplings than in those only subjected to drought (Figure 5c and Supporting Information: Table S2).

Gas exchange variables and the water content of needles were common predictors of mortality rates across the three stress treatments in spruce (Figure 4a,c,e). Defoliation was significant only in the drought‐inoculation treatment, where it was the second most important predictor (Figure 4e). Both drought and pathogen infection caused defoliation levels similar to that recorded for the combined drought‐inoculation treatment after the fourth month (Supporting Information: Figure S6a).

**Figure 6:**
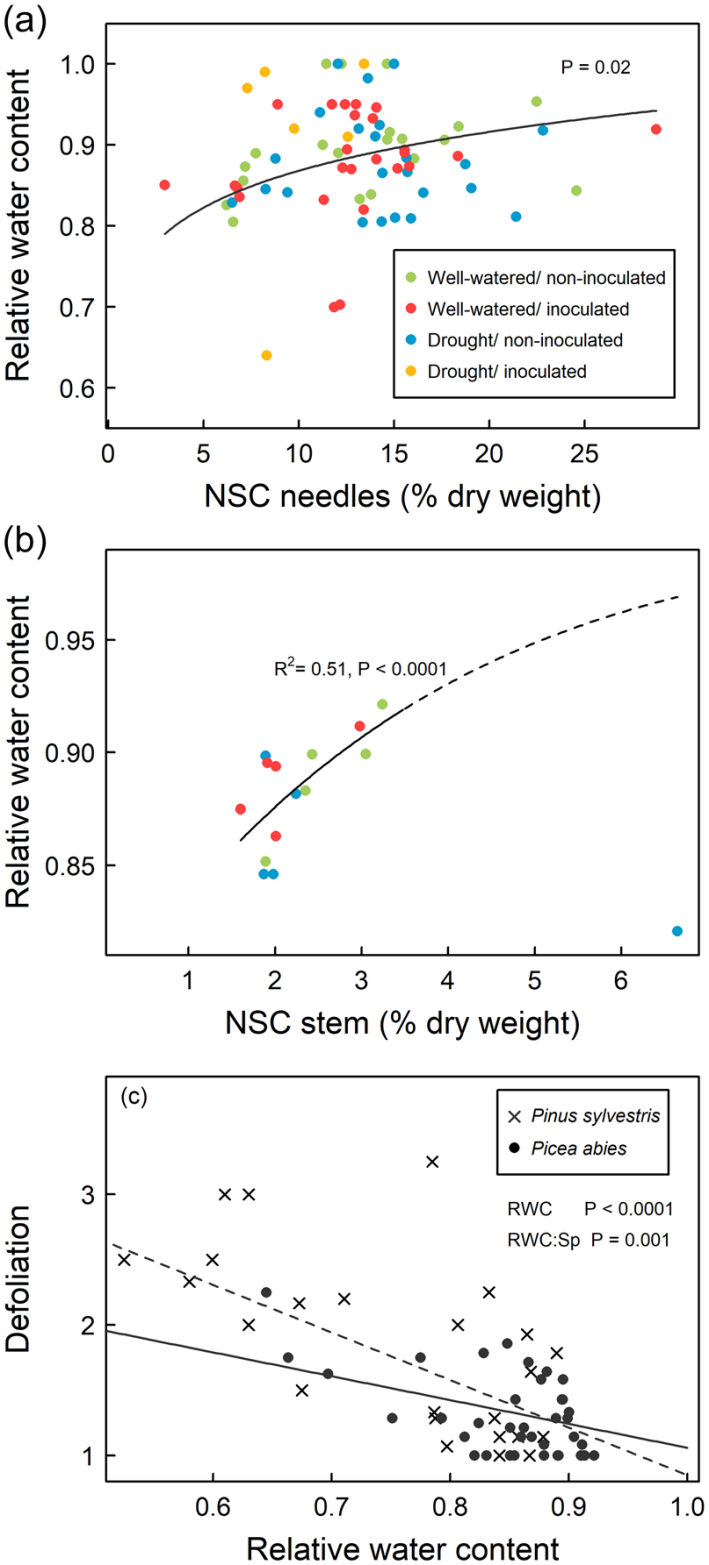
Relationship between needle relative water content and the nonstructural carbohydrate (NSC) concentration of spruce needles (a). Model of beta‐regression linking stem NSC concentration with relative water content in spruce needles (b). Linear model of the relative water content in needles and defoliation using average values at all sampling points; all treatments are included (c). The solid line corresponds to spruce and the dashed line to pine. Defoliation is graded from 0 to 4 (0, no defoliation; 1, 25% defoliated; 2, 50% defoliated; 3, 75% defoliated; 4, completely defoliated).

The effect of *RWC* on defoliation was confirmed by linear models (Figure 6c). In addition, *RWC* was positively correlated with NSC content in needles across treatments (Figure 6a), and with NSC concentrations in stems in surviving spruce (Figure 6b). These correlations point to the coordination of saplings' water and C pools and their defoliation level, which was linked to mortality risk under drought and pathogen infection.

### 3.2 Drought or inoculation alone led to the mortality of Scots pine saplings

Pines subjected to drought or pathogen infection died at a similar rate. The combined drought and inoculation treatment did not increase the mortality risk in pine. We found no differences in terms of survival among saplings that were either inoculated, subjected to drought or both (Figure 1b and Supporting Information: Table S1). Seventy‐five percent of deaths of inoculated pine saplings occurred within the first 3 months of inoculation.

Pathogen infection caused hydraulic impairment in pine. The impact of inoculation on pine physiological variables was similar to that observed for drought and when the two stress treatments were combined. Saplings subjected to any of the three stress treatments showed a similar reduction in stomatal conductance and photosynthesis, as well as more negative midday water potentials than saplings that received the control treatment (Figure 5 and Supporting Information: Table S2). Surprisingly, the starch concentration in needles of inoculated pine saplings was similar to that of noninoculated controls (despite decreased photosynthesis), but higher than that of the drought‐only treatment (Figure 3d and Supporting Information: Table S2). Like in spruce, inoculation under drought conditions did not increase *PLC* compared with that of pine saplings that were only subjected to drought (Supporting Information: Figure S5).

Defoliation was the variable that most strongly increased the mortality risk in pine (Figure 4b,d,f). Before death, the youngest shoots of pine saplings displayed a conspicuous loss of turgor, coupled with a general shedding of older needles.

Like in spruce, variables related to water, the C economy and C uptake were significantly associated with mortality in all treatments, with needle *RWC* being the only variable that was significant across treatments (Figure 4). Mortality rates of inoculated‐only pine were not associated with the NSC content of needles (Figure 4b). By contrast, both inoculated and noninoculated pine saplings subjected to drought, which had lower starch concentrations in needles died faster (Figure 4d,f).

## 4 DISCUSSION

We studied the interactive effect of drought and pathogenic infection on the mortality process of two conifer species. We used *H. annosum* s.s., a necrotrophic fungus that attacks sapwood and causes internal decay and a reduction of functional sapwood. We only found an additive effect of drought and pathogen infection in spruce, where the combination of these two stressors accelerated death compared with that observed for each individual stressor (i.e., our results support Hypothesis [i] for one of the study species). Spruce saplings suffered from decreased C reserves and increased hydraulic stress when subjected to both drought and pathogen infection. Specifically, pathogen infection decreased the concentration of starch in the needles of saplings subjected to drought, a variable linked to mortality in our survival analysis.

### 4.1 Drought–pathogen interactions in spruce

**Figure 7:**
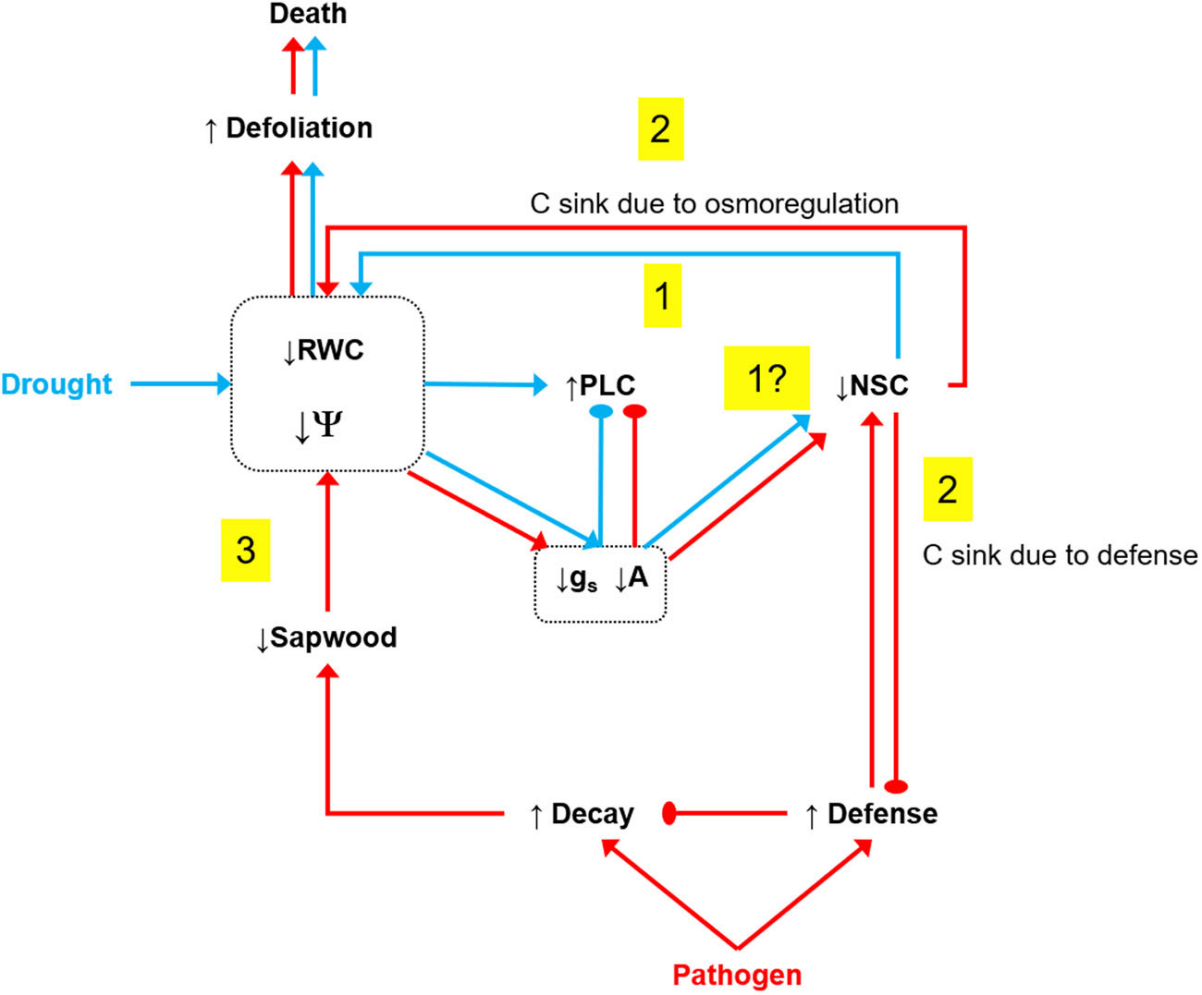
Diagram of the mechanisms of tree mortality induced by the concurrence of drought and pathogen infection in spruce. Three mechanisms are highlighted: (1) drought affects carbon (C) reserves and impairs the capacity of trees to build up defence barriers to compartmentalize the pathogen; (2) pathogen infection triggers a defence response, which creates a C sink, reducing C availability for maintaining needle turgor; and (3) the pathogen reduces functional sapwood and disturbs water transport and storage in the presence of drought. *A*, net assimilation rate; *g*_s_, stomatal conductance; *PLC*, percentage loss of conductivity; NSC, nonstructural carbohydrates; *RWC*, needle relative water content; *Ψ*, water potential.

Our results support two of our three hypothesized mechanisms by which the additive effect of drought and infection occurs in spruce (Figure 7): defence needs associated with pathogenic infection reduce the amount of C available to cope with drought, which eventually impairs the water status (mechanism 2); and pathogen infection affects hydraulic performance through a reduction in the sapwood cross‐sectional area (mechanism 3). Firstly, we showed that building a reaction zone was needed to contain pathogen colonization and survive given that survivors had a larger reaction zone than dead spruce saplings. The larger the reaction zone size, the lower the SS concentration found at the site of infection. This, together with the reduced C content in needles of inoculated spruce, suggests that the rate of C fixation in the reaction zone was faster than the rate of C replenishment at the site of infection via translocation from needles. The C needs at the site of infection may then compete with other C sinks such as osmoregulation to avoid dehydration caused by drought. The low NSC concentration in the stem and needles was linked to the low *RWC* in needles. These results point to a central role of plant water content (Martinez‐Vilalta et al., 2019; Sapes et al., 2019), which, coupled with C sink demands, would determine defoliation levels and subsequent mortality under drought and pathogen attack. Secondly, pathogen colonization of the inner part of the stem reduced functional sapwood, which aggravated the hydraulic impairment caused by drought, resulting in more negative water potential values than in noninoculated saplings subjected to drought. Finally, we did not find any direct drought effects on C reserves linked to spruce survival (mechanism 1, Figure 7). However, the lowest photosynthetic rates were recorded in drought‐inoculated spruce, which partly supports our first hypothesized mechanism (i.e., drought impairing C uptake, and hence, limiting the amount of C available to build up defence barriers against the pathogen).

Maintaining needle turgor to prevent desiccation is probably the highest priority of trees under drought stress (Martinez‐Vilalta et al., 2019; Sapes et al., 2019). Accordingly, *RWC* values were relatively stable across time and treatments in our experiment. Reduced *RWC* resulted in an increased probability of death and was related to increased defoliation across treatments and species. Although defoliation may be the result of an acclimation response to drought and pathogen infection to improve sapling performance (Martínez‐Vilalta et al., 2009; Papú et al., 2021), severe defoliation signals extreme stress responses associated with an increased mortality risk (Poyatos et al., 2013). In particular, defoliation implies lowered C assimilation (and eventually lower C reserves) due to reduced photosynthetically active tissue and may lead to an increase in energy costs to maintain cell turgor under more negative osmotic pressures (Galiano et al., 2011; Salmon et al., 2015; Sapes et al., 2019). Purely pathogen‐induced hydraulic impairment and defoliation was evident in pine; however, this was only apparent in combination with drought stress in spruce. Indeed, pathogen colonization of the sapwood, in the absence of drought, did not impose hydraulic constraints on spruce given that water potential, *RWC* and *PLC* in inoculated‐only spruce saplings were similar to those of controls. This is consistent with previously reported sapwood redundancy, by which a 50% reduction in sapwood area caused relatively small reductions in leaf conductance and had no significant impact on shoot water potential in Norway spruce (Dietrich et al., 2018; see also Körner, 2019). However, under drought conditions, pathogen‐induced loss of sapwood had an impact on water potential, aggravating drought‐induced hydraulic dysfunction.

We showed that a decrease in available C at the site of inoculation was associated with the saplings' defence response, particularly with the size of the reaction zone. In addition, C uptake was not reduced in inoculated‐only spruce even though lower starch and SS concentrations in needles were associated with the mortality of inoculated saplings. This result suggests that the reaction zone formed to compartmentalize the pathogen increases the requirement for C, which needs to be fulfilled. This is in line with the fact that reaction zones are C‐enriched compared to healthy sapwood (10% of C content increase reported for *Ophiostoma brunneo ciliatum* by Guérard et al., 2007), with the activation of genes that allocate C to secondary metabolism (Oliva et al., 2015), and with a density increment of ca. 15% of the reaction zone versus sapwood under *Heterobasidion* infection (Oliva et al., 2011). Spruce saplings surviving inoculation had both a higher NSC content in needles and twigs and a larger reaction zone than those that died, which implies that their C budget allowed for both an adequate defence response and the maintenance of C reserves. When infection coincides with drought, lower rates of photosynthesis and the requirement of NSC for osmoregulation in needles and shoots also contribute to the overall C demand. However, other mechanisms could also have limited the C supply to the site of infection. NSC transport could have been impaired by the necrosis of the phloem by the pathogen. Our study does not enable us to evaluate the impact of drought and pathogen infection on C transport in the phloem. However, lesions in the phloem were particularly large in spruce subjected to both drought and inoculation, as previously reported for other sapwood‐ and phloem‐infecting fungi that cause larger phloem lesions under drought conditions (Netherer et al., 2016; Schoeneweiss, 1975, 1983; Terhonen et al., 2019). Drought has also been suggested to cause phloem transport failure (Sevanto, 2014). Thus, the possible impairment of C transport in the phloem under drought and pathogen infection warrants further research.

### 4.2 Differences between species

Pathogen inoculation alone had more severe effects on pine than on spruce. The disease produced in our experiment corresponded well with how pine and spruce interact with the pathogen in nature, where spruce trees typically suffer from internal decay, whereas pines usually succumb as a result of cambial death and fungal growth in the outer sapwood (Korhonen & Stenlid, 1998). Observed differences may be due to the capacity of spruce to create a thick reaction zone around the decay column, which was not seen in pine. However, in our experiment, we deliberately introduced the pathogen into the sapwood, thereby eliminating bark and phloem defence barriers. These barriers might be more important for defending pine against *Heterobasidion* infection than they are for spruce; hence, the observed mortality differences between species may be partly due to our experimental setup. Although pine is the preferred host for *H. annosum* s.s., it is able to infect spruce (Garbelotto & Gonthier, 2013). This may explain the high level of aggressivity observed in pine and the short time needed to cause mortality in our experiment. We argue that the rapid rate of mortality of inoculated pine did not allow for the expression of the interaction between *Heterobasidion* infection and drought. Lower inoculum loads of the pathogen in the field, compared with mycelial plugs inserted directly into the sapwood of saplings, may result in a slower mortality process due to infection in the field, which could be amplified by drought. In the case of spruce, *H. parviporum* can be more aggressive than *H. annosum* s.s. on Norway spruce (Garbelotto & Gonthier, 2013; Oliva et al., 2011); therefore, drought is likely to have a greater impact on the pathogenic infection and host mortality in trees infected with *H. parviporum*. Accordingly, Terhonen et al. (2019) reported larger drought‐induced increases in lesion dimensions in spruce infected by *H. parviporum* rather than by *H. annosum* s.s.

The effect of infection also led to a different impact on the gas exchange of the two host species. In spruce, there was neither stomatal closure nor photosynthetic down‐regulation under *H. annosum* s.s. infection compared with that of the control group across the experiment. By contrast, in pine, fungal infection resulted in a significant decrease in stomatal conductance and photosynthetic rate. The decrease in gas exchange has been reported previously as a host response to canker diseases, which mainly impact vascular cambium and phloem tissue (da Silva et al., 2018; Ghanbary et al., 2020; Hossain et al., 2018; Li et al., 2019; Madmony et al., 2018; Xing et al., 2020). The differential stomatal response to pathogenic infection between the two host species should be investigated in future work to clarify the relative role of the reduction in water potential, hormonal signals and potential hormonal crosstalk (defence and drought stress) in stomatal control (Beguerisse‐Diaz et al., 2012). Despite the differences in the physiological response to pathogen inoculation between the two species, they presented similar mortality rates when pathogen infection occurred under drought conditions.

Most of the spruce mortality events associated with the two inoculation treatments took place after the dormancy period. This was particularly evident for the well‐watered inoculated spruce saplings: none died before dormancy, whereas 40% of deaths occurred after dormancy. A similar pattern was observed for spruce subjected to drought and inoculation, with 30% and 60% of the total deaths taking place before and after dormancy, respectively. Hibernation conditions in our experiment resembled a mild winter (above‐zero temperatures and a lack of light) in the boreal zone. We speculate that during dormancy, the fungus might have taken advantage of the saplings' inactive defence system and continued to grow in the sapwood, contributing to the reduction of functional sapwood. *Heterobasidion annosum* s.s. is able to grow at temperatures as low as 2°C (1.1 mm/day at 5°C; Taubert, 2008). The temperature in our experimental dormancy was never lower than 5°C, and therefore, likely permitted fungal growth in inoculated saplings. Thus, expansion of *H. annosum* might have happened during dormancy, leading to the high percentages of decayed sapwood (94% in inoculated saplings that died during the first month following dormancy) upon the reestablishment of light and vegetative activity. The interaction between drought, the pathogen and mild winters may have important implications under ongoing climate change because a future warmer climate would increase the likelihood of these three factors co‐occurring in many areas, including boreal forests. However, we reached these conclusions based on our study of saplings and caution should be taken when extrapolating them to mature trees, whose larger dimensions may protect them from lethal reductions of functional sapwood.

## 5 CONCLUSIONS

Our results suggest that mortality due to simultaneous drought and pathogen infection is linked to the limited availability of C, which is needed to respond to the two main C sinks created by the stresses: the defence response at the site of infection and osmoregulation to maintain cell turgor in needles, leading to impaired water status. The additive effect of the two stressors was significant in spruce, which showed an accelerated death rate compared with saplings subjected to only one of the stressors. By contrast, pine saplings died at a similar rate when experiencing either drought or pathogen infection or both stressors. In our pathosystem, maintaining turgor was associated with a higher availability of NSC, presumably impairing the sapling's capacity to allocate NSC to build a reaction zone and contain the pathogen. Although this conclusion may differ in other pathogen–host trophic interactions affecting different plant organs, we argue that pathogens can exploit C allocation priorities under stress and increase their capacity to attack vital plant organs. Similarly, our results show how pathogens can benefit from host dormancy to cause mortality.

We encourage empirical studies of trophic interactions other than necrotrophy to fully elucidate how co‐occurring drought and pathogen infection can lead to tree mortality. The incorporation of both pathogen‐induced and drought‐induced mortality mechanisms in models has the potential to improve our capacity to forecast forest dynamics under climate change (Trugman et al., 2021). A way to capitalize on these results in tree mortality modelling could be the inclusion of an acceleration factor in death rates, if drought events coincide with the presence of necrotrophic pathogens infecting vascular tissues. However, we recognize that pathogen–drought interactions are complex and multidimensional. Drought can have simultaneous, non‐linear impacts on both host and pathogen populations (Dudney et al., 2021), and therefore, integrative research including all these impacts is needed to improve mechanistic tree mortality modelling.

## Supporting information

Supporting information.

## Data Availability

The data that support the findings of this study are available from the corresponding author upon reasonable request.
