# Supplementary material for: Interaction of drought‐ and pathogen‐induced mortality in Norway spruce and Scots pine
Source: Plant Cell Environ. 2022 May 31;45(8):2292–305. doi: 10.1111/pce.14360 (PMC9546048; doi:10.1111/pce.14360)
Supplement: Supplementary file 1 — Supporting information. [file PCE-45-2292-s001.docx]

Supplementary material


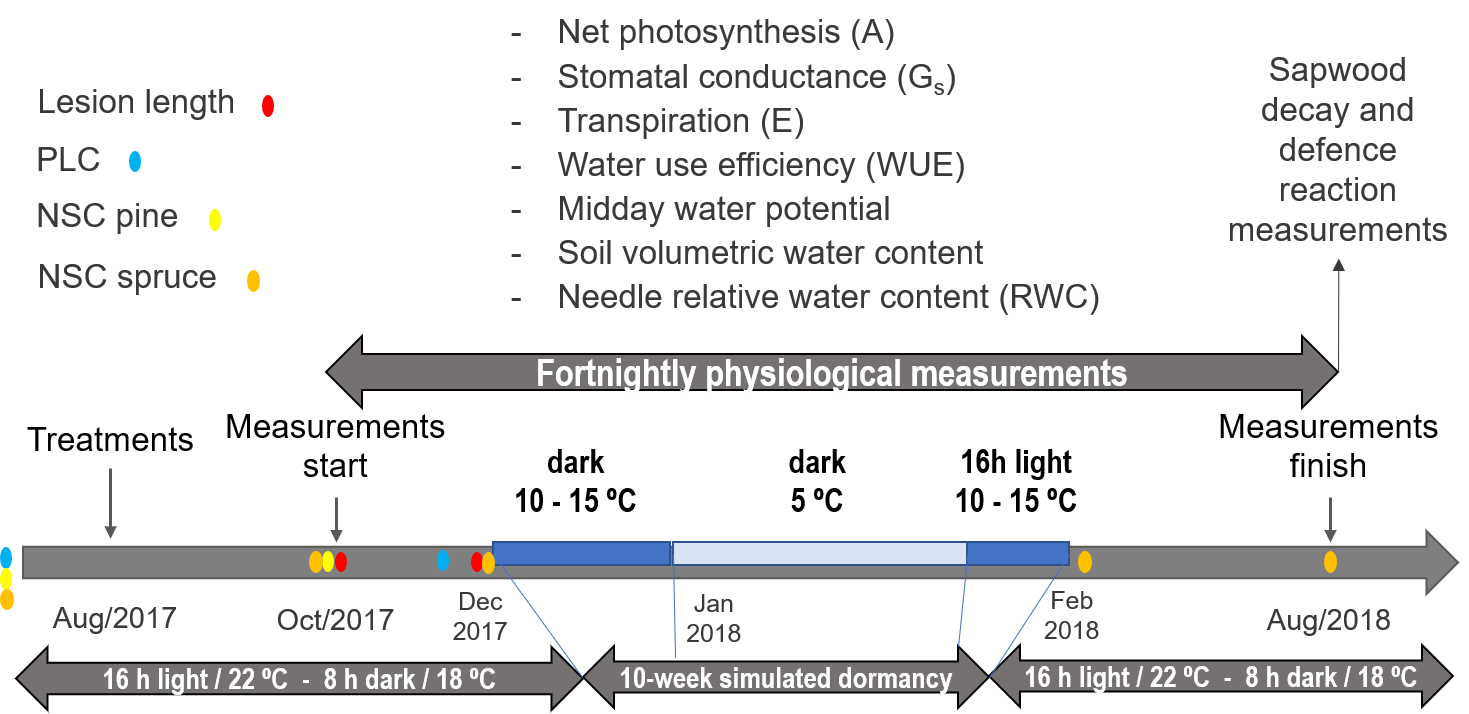


Figure S1. Flowchart illustrating the experimental design, measurements and dates.


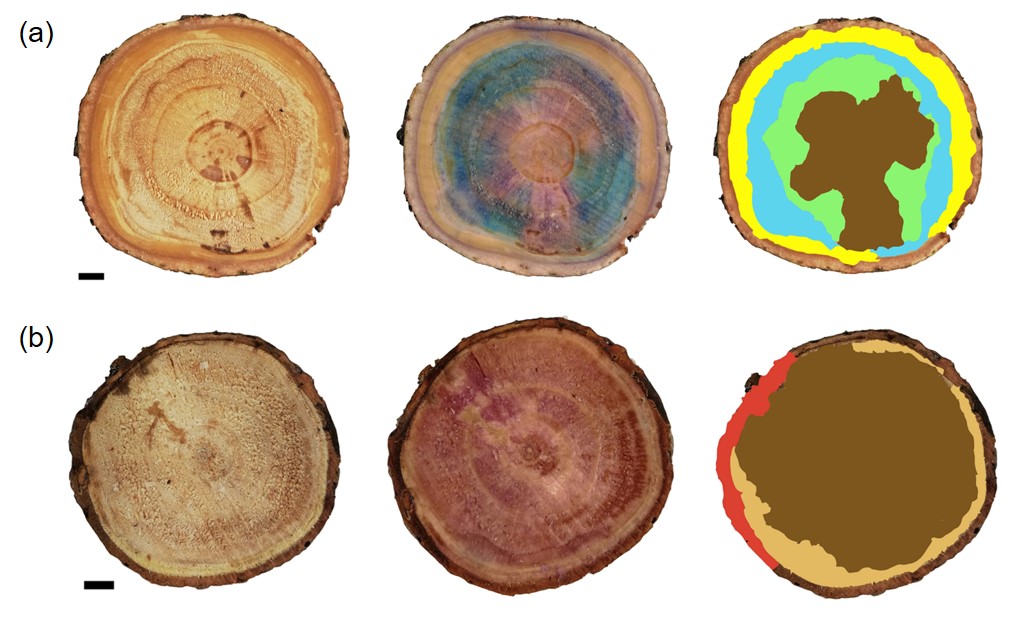


Figure S2. Cross sections of representative well-watered/inoculated saplings, which either survived (a) or died (b). In the first column, fresh-cut cross sections. In the second column, cross section after spraying with 2,6 dichlorophenolindophenol, a pH indicator to detect elevated pH typical of the defence reaction zone. In the last column, classification into functional sapwood (yellow), active reaction zone (blue), old reaction zone (green), intermediate (light marron) and advanced (dark marron) decayed sapwood, and necrosis (red). Bars = 5 mm.


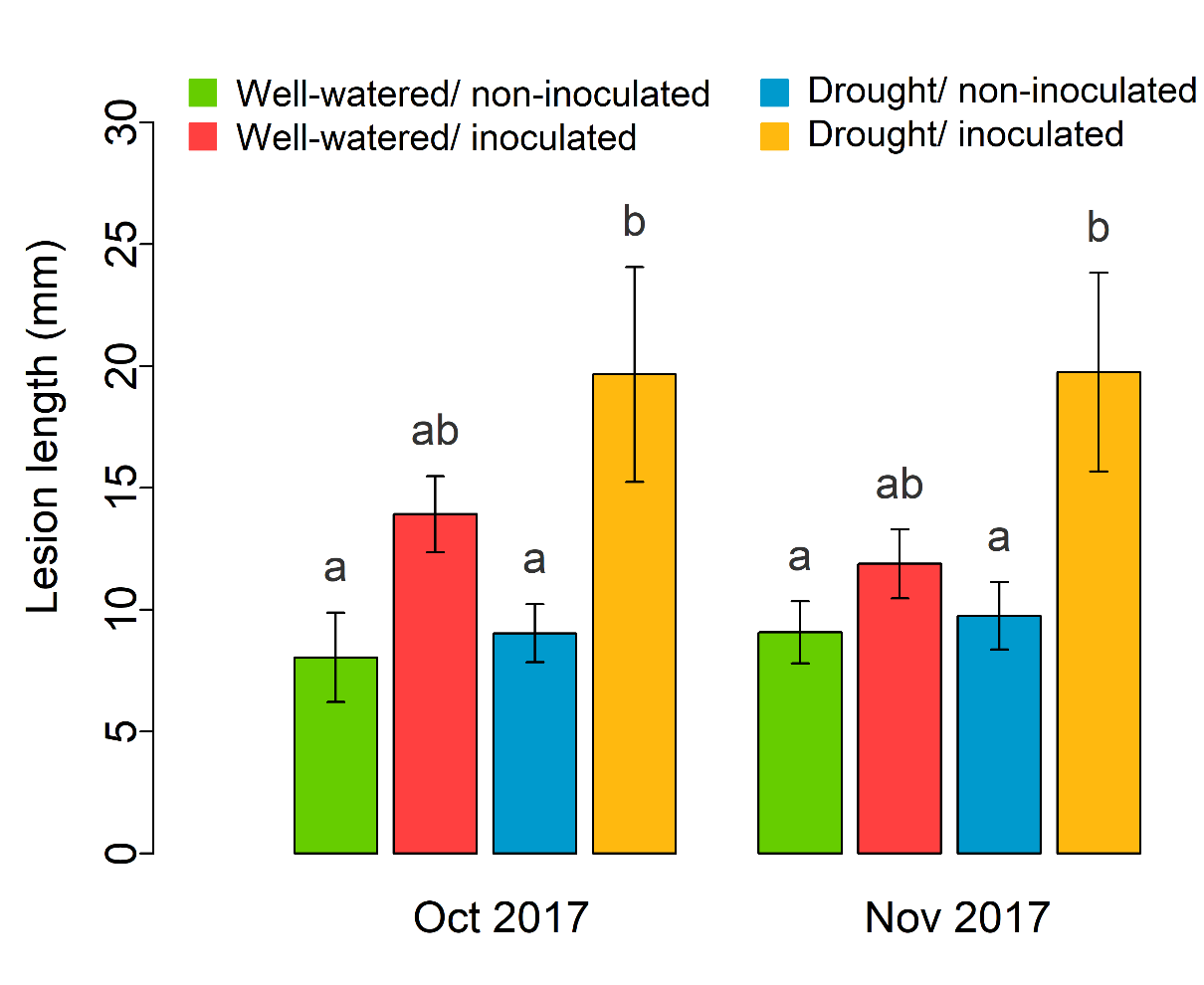


Figure S3. Lesion length (mm) estimated as the horizontal radius of the ellipse-shaped lesion in the inner bark of spruce saplings. Different letters indicate statistically significant differences (P = 0.05) between treatments within species on a multiple comparison procedure using Tukey.


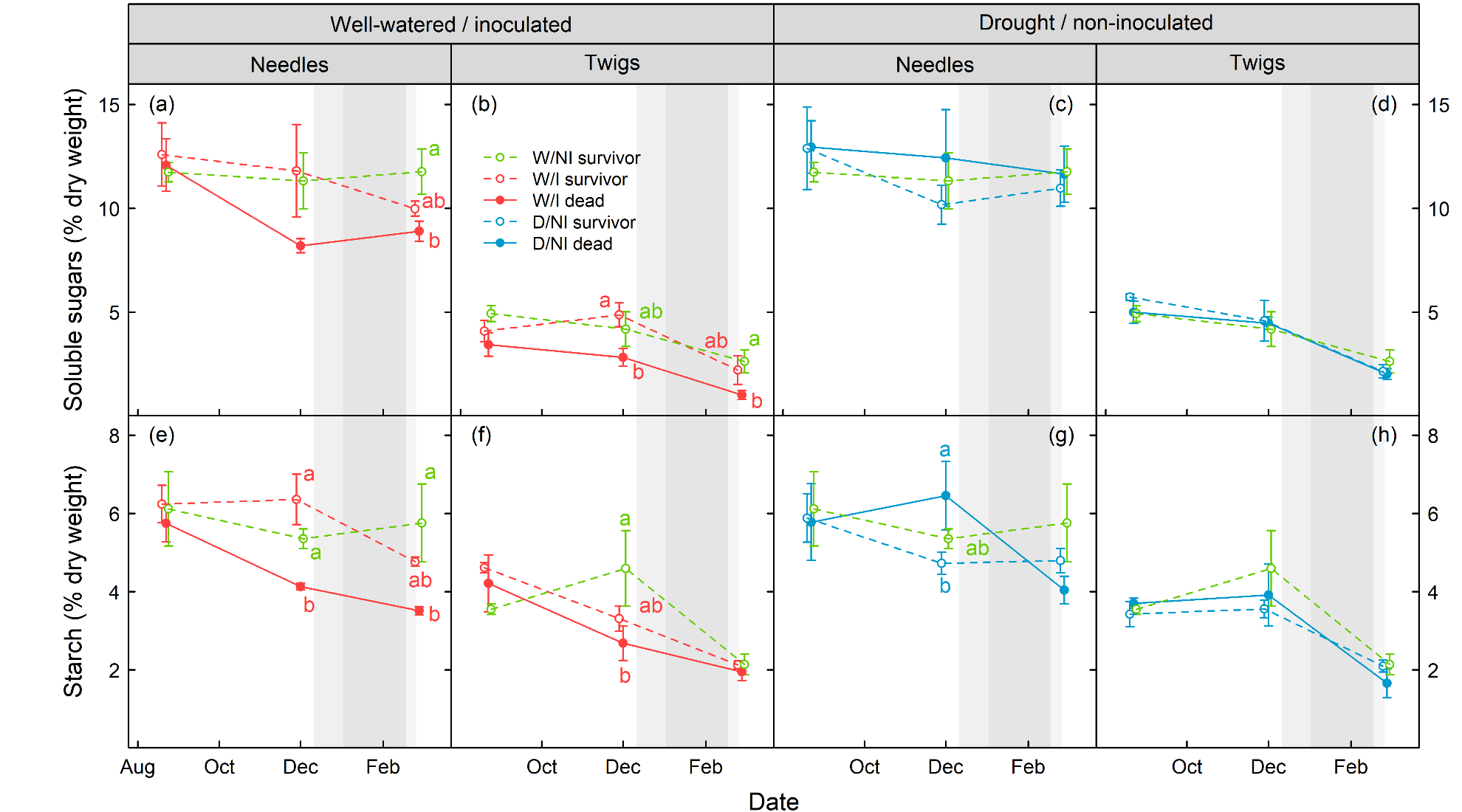


Figure S4. Soluble sugars (a-d) and starch (e-h) concentration in spruce needles and twigs of well-watered/inoculated survivors vs saplings that eventually died (a-b, e-f), and drought/non-inoculated survivors vs saplings that eventually died (c-d, g-h). Values for well-watered/non-inoculated survivors are included as reference. Different letters indicate statistically significant differences (P < 0.05) between treatments within species on a multiple comparison procedure using Tukey, given by linear mixed models.


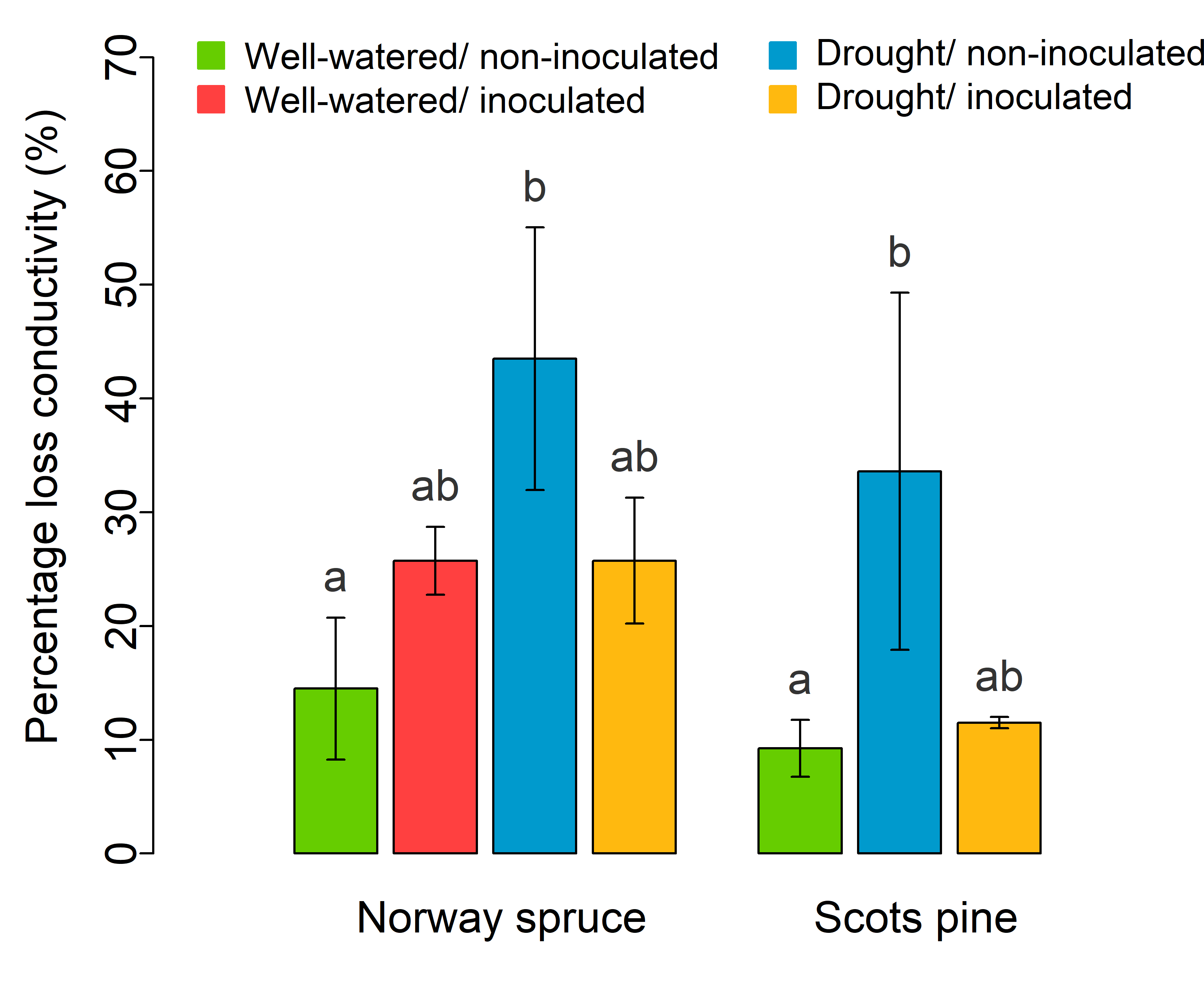


Figure S5. Percentage loss of conductivity (PLC, %) for *Picea abies* (Norway spruce) and *Pinus sylvestris* (Scots pine). PLC was measured the 1^st^ of December 2017, i.e. 3.5 months after treatments.


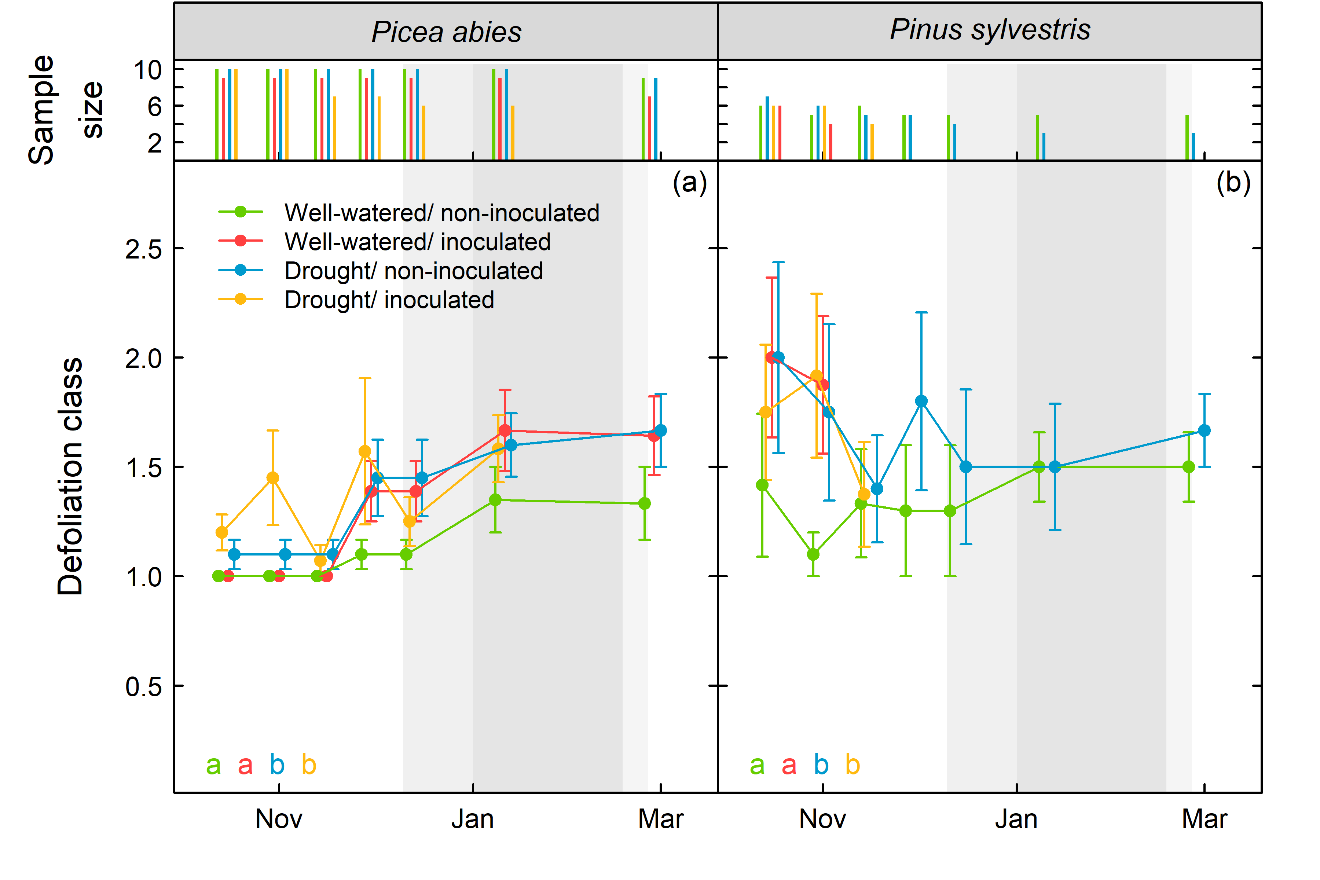


Figure S6. Defoliation levels graded from 0 to 4 for each treatment and species (0, no defoliated; 1, 25% defoliated; 2, 50% defoliated; 3, 75% defoliated; 4, completely defoliated).. Different letters indicate statistically significant differences (P < 0.05) between treatments within species given by the linear mixed models on a multiple comparison procedure using Tukey. The sample size (number of saplings) for each sampling date is indicated on the top panels.

Table S1. Predicted survival time (days after treatments) from the accelerated failure time parametric model for each treatment and species. Different uppercase letters indicate statistically significant differences (P < 0.05) between treatments within species on a multiple comparison procedure using Tukey; lowercase letters indicate differences between species within treatments. Numbers in parenthesis indicate standard errors. W/NI: well-watered/non-inoculated; W/I: well-watered/inoculated; D/NI: drought/non-inoculated; D/I: drought/inoculated.

|  | Survival time | |
| --- | --- | --- |
|  | ***Pinus sylvestris*** | ***Picea abies*** |
| W/NI | 365 (138) ^A a^ | 594 (203) ^A a^ |
| W/I | 97 (29) ^B a^ | 358 (96) ^AB b^ |
| D/NI | 135 (38) ^AB a^ | 409 (106) ^A b^ |
| D/I | 97 (27) ^B a^ | 149 (31) ^B a^ |

Table S2. Least square means predicted of the physiological variables for each treatment as given by the linear mixed models. Different letters indicate statistically significant differences (P < 0.05) between treatments within species on a multiple comparison procedure using Tukey. Numbers in parenthesis indicate standard errors. W/NI: well-watered/non-inoculated; W/I: well-watered/inoculated; D/NI: drought/non-inoculated; D/I: drought/inoculated. *RWC*: relative water content; *Ψ_md_*: midday water potential (MPa); *g_s_*: stomatal conductance (mmol m^-2^ s^-1^); *A*: net photosynthesis (µmol m^-2^ s^-1^); *SS*: soluble sugars (%). Defoliation is graded from 0 to 4.

|  | *Picea abies* | | | | *Pinus sylvestris* | | | |
| --- | --- | --- | --- | --- | --- | --- | --- | --- |
|  | **W/NI** | **W/I** | **D/NI** | **D/I** | **W/NI** | **W/I** | **D/NI** | **D/I** |
| *RWC* | 0.88 (0.01) ^a^ | 0.89 (0.01) ^a^ | 0.85 (0.01) ^b^ | 0.83 (0.01) ^b^ | 0.85 (0.01) ^a^ | 0.79 (0.03) ^b^ | 0.83 (0.02) ^a^ | 0.77 (0.03) ^b^ |
| *Ψ_md_* | -1.50 (0.11) ^a^ | -1.54 (0.11) ^a^ | -1.78 (0.11) ^b^ | -2.01 (0.13) ^c^ | -0.92 (0.19) ^a^ | -1.35 (0.23) ^b^ | -1.26 (0.20) ^b^ | -1.31 (0.22) ^b^ |
| *g_s_* | 32.8 (4.8) ^a^ | 34.6 (4.8) ^a^ | 24.4 (4.6) ^b^ | 26.2 (4.9) ^b^ | 40.9 (5.3) ^a^ | 13.4 (5.4) ^b^ | 14.0 (4.4) ^b^ | 13.6 (5.5) ^b^ |
| *A* | 3.58 (0.42) ^a^ | 3.73 (0.43) ^ab^ | 2.57 (0.41) ^b^ | 1.51 (0.44) ^c^ | 3.51 (0.39) ^a^ | 1.86 (0.60) ^b^ | 1.64 (0.37) ^b^ | 2.18 (0.63) ^b^ |
| *Defoliation* | 1.17 (0.08) ^a^ | 1.19 (0.08) ^a^ | 1.33 (0.08) ^b^ | 1.35 (0.08) ^b^ | 1.38 (0.09) ^a^ | 1.61 (0.14) ^a^ | 1.67 (0.11) ^b^ | 1.89 (0.14) ^b^ |
| *SS needles* | 10.14 (1.33) ^a^ | 8.89 (1.30) ^b^ | 9.66 (1.31) ^a^ | 8.41 (1.35) ^b^ | 3.78 (0.36) ^a^ | 3.78 (0.39) ^a^ | 4.14 (0.36) ^a^ | 4.14 (0.36) ^a^ |
| *Starch needles* | 5.65 (0.42) ^a^ | 4.62 (0.31) ^b^ | 5.05 (0.34) ^a^ | 4.01 (0.37) ^b^ | 3.92 (0.15) ^a^ | 4.03 (0.16) ^a^ | 3.35 (0.15) ^b^ | 3.46 (0.15) ^b^ |

Table S3. Least square means predicted from the beta regression analysis of soluble sugars and starch content (% dry weight) of needles and twigs of *Picea abies* before dormancy (3.5 months after treatments, 1 December 2017), and after dormancy (28 February 2018) for survivors and saplings that died in well-watered/inoculated (W/I) and drought/non-inoculated (D/NI) treatments. Well-watered/non-inoculated (W/NI) survivors were included in the analyses as a control level. Numbers in parenthesis indicate standard errors. Different letters indicate statistically significant differences (P < 0.05) between groups on a multiple comparison procedure using Tukey.

|  | SOLUBLE SUGARS IN NEEDLES | | | | | |
| --- | --- | --- | --- | --- | --- | --- |
|  | **W/I - W/NI** | | | **D/NI - W/NI** | | |
|  | **W/NI - survivor** | **W/I - survivor** | **W/I - dead** | **W/NI - survivor** | **D/NI - survivor** | **D/NI - dead** |
| Before dormancy | 11.4 (1.2) ^a^ | 11.6 (1.3) ^a^ | 8.5 (1.2) ^a^ | 10.4 (1.2) ^a^ | 12.1 (1.5) ^a^ | 11.4 (1.3) ^a^ |
| After dormancy | 11.7 (0.7) ^a^ | 10.1 (0.6) ^ab^ | 9.0 (0.6) ^b^ | 11.0 (0.9) ^a^ | 11.6 (1.0) ^a^ | 11.8 (0.9) ^a^ |
|  | **SOLUBLE SUGARS IN TWIGS** | | | | | |
| Before dormancy | 4.1 (0.6) ^ab^ | 4.9 (0.6) ^a^ | 2.9 (0.5) ^b^ | 4.4 (0.7) ^a^ | 4.7 (0.8) ^a^ | 4.2 (0.7) ^a^ |
| After dormancy | 2.6 (0.5) ^a^ | 2.1 (0.4) ^ab^ | 1.2 (0.3) ^b^ | 2.2 (0.3) ^a^ | 2.1 (0.4) ^a^ | 2.5 (0.4) ^a^ |
|  | **STARCH IN NEEDLES** | | | | | |
| Before dormancy | 5.4 (0.4) ^a^ | 6.3 (0.4) ^a^ | 4.2 (0.3) ^b^ | 4.8 (0.4) ^a^ | 6.4 (0.5) ^ab^ | 5.4 (0.4) ^b^ |
| After dormancy | 5.6 (0.5) ^a^ | 4.9 (0.5) ^ab^ | 3.6 (0.5) ^b^ | 4.9 (0.5) ^a^ | 4.1 (0.5) ^a^ | 5.6 (0.6) ^a^ |
|  | **STARCH IN TWIGS** | | | | | |
| Before dormancy | 4.5 (0.6) ^a^ | 3.4 (0.5) ^ab^ | 2.7 (0.5) ^b^ | 3.7 (0.5) ^a^ | 3.9 (0.6) ^a^ | 4.5 (0.6) ^a^ |
| After dormancy | 2.1 (0.2) ^a^ | 2.2 (0.2) ^a^ | 1.9 (0.2) ^a^ | 2.1 (0.) ^a^ | 1.6 (0.2) ^a^ | 2.1 (0.2) ^a^ |
